# Toward a Unified Neuroimmune Framework for Infection-Associated Psychiatric Disorders

**DOI:** 10.3390/diseases14080290

**Published:** 2026-08-11

**Authors:** Manuela Arbune, Pantelie Nicolcescu, Anamaria Ciubara, Pompiliu Mircea Bogdan, Constantin-Marinel Vlase, Anca-Adriana Arbune

**Affiliations:** 1 Faculty of Medicine and Pharmacy, Research Center in the Medical-Pharmaceutical Field, “Dunarea de Jos” University of Galați, 800008 Galati, Romania; manuela.arbune@ugal.ro (M.A.); anamaria.ciubara@ugal.ro (A.C.); 2 Doctoral School of Biomedical Sciences, Faculty of Medicine and Pharmacy, Research Center in the Medical-Pharmaceutical Field, “Dunărea de Jos” University of Galați, 800008 Galati, Romania; pompiliu.bogdan@ugal.ro; 3 Emergency Military Hospital Galați Dr. Aristide Serfioti, 800150 Galati, Romania; constantin.vlase@ugal.ro; 4 Multidisciplinary Integrated Center of Dermatological Interface Research (MIC-DIR) Research Unit, “Dunarea de Jos” University of Galați, 800008 Galati, Romania; aarbune@spital-obregia.ro; 5 Department of Stress Prevention and Research, “Prof. Dr. Al. Obregia” Psychiatry Clinical Hospital, 041914 Bucharest, Romania

**Keywords:** infectious disease, neuroinflammation, psychiatric disorder, blood–brain-barrier, biomarker, neurotrophic factors

## Abstract

Background/Objectives: Neuroinflammation is increasingly recognized as a key mechanism linking infectious diseases with psychiatric disorders through interactions between peripheral immune activation, metabolic pathways, and brain network alterations. This review aimed to synthesize current evidence on the neuroimmune mechanisms and biomarkers underlying infection-associated psychiatric disorders. Methods: A narrative literature review structured according to the SANRA (Scale for the Assessment of Narrative Review Articles) criteria was conducted using the Web of Science Core Collection, PubMed/MEDLINE, Scopus and PsycINFO databases. Boolean search strategies identified studies investigating neuroinflammatory biomarkers, neuroimmune mechanisms, and psychiatric outcomes associated with infectious diseases. The search (January 2022–30 June 2026) included 76 studies in the final qualitative analyses. Results: The reviewed evidence consistently identified inflammatory cytokines and chemokines, complement proteins, blood–brain barrier markers, glial activation biomarkers, neuroaxonal injury markers, kynurenine pathway metabolites, neurotrophic factors, and neuroimaging markers as complementary indicators of infection-induced neuroimmune dysfunction. Across diverse bacterial, viral, parasitic, and systemic infections, these mechanisms converged on peripheral immune activation, blood–brain barrier disruption, microglial activation, kynurenine pathway dysregulation, impaired neurotrophic signaling, synaptic dysfunction, and altered brain network connectivity, contributing to depression, anxiety, psychosis, cognitive impairment, and fatigue. Based on these findings, a unified neuroimmune model integrating peripheral and central mechanisms is proposed. Conclusions: Neuroinflammation emerges as a shared biological pathway linking infections with transdiagnostic psychiatric phenotypes. Although no single biomarker currently demonstrates sufficient diagnostic specificity, integrated multimodal biomarker panels may improve biological stratification, facilitate earlier identification of high-risk patients, and support the development of mechanism-based precision approaches—including candidate anti-inflammatory pharmacological strategies currently under clinical investigation—for infection-associated psychiatric disorders.

## 1. Introduction

Mental disorders constitute one of the leading burdens of public health, with increasing prevalence worldwide. According to a World Health Organization (WHO) report, in 2021, approximately one in seven individuals were living with a mental disorder [1].

Infectious diseases have accompanied human history, shaping the evolution of the species both through physical health and by generating inflammatory responses involved in the pathogenesis of various mental disorders. The relationship between infectious diseases and mental health has long been recognized as bidirectional, involving complex and still incompletely understood mechanisms.

Infectious diseases have been associated with a wide range of psychiatric symptoms, including mood disorders, cognitive impairments, and neurocognitive disturbances. Relevant research has shown that mental disorders increase susceptibility to infections, while infections also have a significant impact on mental health [2].

Infections can exacerbate or trigger mental disorders, leading to significant morbidity and mortality. Individuals with severe mental illnesses have a 2.7-fold higher risk of death from infectious diseases, and the risk of death from respiratory infections is more than three times higher compared to the general population [3].

A Mendelian randomization study demonstrated a causal relationship between anxiety, depression, and sleep disorders and the risk of developing infectious diseases. However, no causal association was supported for other mental disorders or states of nervousness [4].

Okobi et al. (2024) found that, in a nationwide population-based study conducted in Denmark, hospitalization for severe infections was associated with an approximately 63% increased risk of subsequently developing affective disorders [2]. The relationship between mental disorders and infectious diseases is bidirectional, with mental disorders acting both as a consequence of infection and as a risk factor for infectious diseases, thereby complicating clinical management. Infectious diseases may affect mental health through anxiety, depression, stigma, impacts on identity, and reduced quality of life, whereas mental health conditions influence risk and protective behaviors related to infections, as well as recovery following infectious illness. In addition, individuals with severe mental disorders have reduced access compared to the general population to high-quality healthcare services, preventive care, screening programs, and management of comorbidities, including infectious diseases [5]. Stigma and discrimination remain major barriers to healthcare access for individuals with severe mental disorders, as highlighted by the Lancet Commission on ending stigma and discrimination in mental health [6]. Neuroinflammatory biomarkers are recognized as key mediators in the interaction between mental health disorders and infections, providing insights into pathophysiological mechanisms and potential therapeutic targets [7]. Both central biomarkers (such as cerebrospinal fluid cytokines and microglial activation) and peripheral biomarkers (such as blood cytokines and acute-phase proteins) have been implicated in psychiatric disorders including depression, psychosis, and bipolar disorder, particularly in the context of concurrent or preceding infections [8,9].

The identification of such biomarkers is essential for early diagnosis, personalized intervention, and monitoring of disease progression or treatment response [10,11]. Although numerous reviews have examined the neuropsychiatric consequences of individual infectious diseases, the current literature remains fragmented across specific pathogens, psychiatric disorders, and isolated neuroimmune mechanisms, limiting the development of a comprehensive mechanistic perspective. To our knowledge, no previous review has systematically integrated the full spectrum of neuroinflammatory biomarkers across bacterial, viral, fungal, parasitic, and systemic infections while proposing a unified transdiagnostic neuroimmune framework linking infection-associated psychiatric disorders.

This narrative review aims to provide a critical synthesis of current knowledge regarding the pathogenic mechanisms and neuroinflammatory biomarkers involved in the association between infectious diseases and mental disorders.

**Figure 1:**
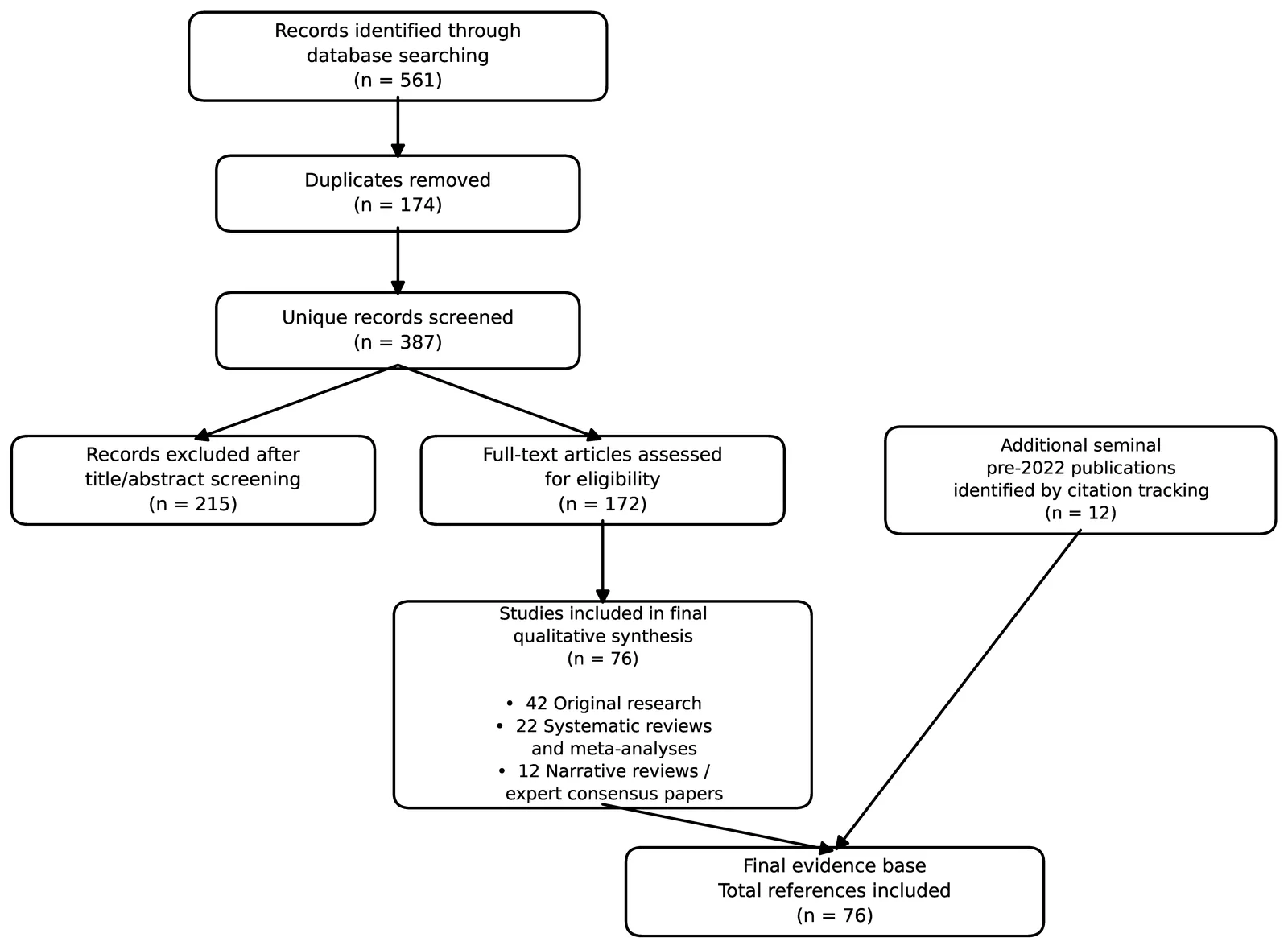
Study selection flow diagram (structured narrative review).

Overview of the Unified Neuroimmune Framework. To improve conceptual coherence, this review is organized around a single Unified Neuroimmune Framework, introduced here and used as the structural backbone of the entire manuscript rather than presented only in the Discussion Section. The framework proposes a sequential, though interacting, cascade: (i) infection-triggered peripheral immune activation (Section 3.1); (ii) gut dysbiosis and blood–brain barrier (BBB) dysfunction enabling peripheral-to-central signal transfer (Section 3.2); (iii) direct or indirect central nervous system invasion and neuroinflammation (Section 3.3); (iv) a heterogeneous spectrum of neuropsychiatric manifestations, illustrated through representative infectious models and the paradigmatic cases of Neuro-COVID and HIV-associated neurocognitive disorder (Section 3.4); (v) reconceptualization of psychiatric disorders as immune-mediated phenotypes, including impaired neurotrophic signaling as a downstream consequence of sustained inflammation (Section 3.5); (vi) systemic pathophysiological amplifiers of immune dysfunction, including HPA-axis dysregulation, autonomic imbalance, immunosenescence, and prenatal immune programming (Section 3.6); and (vii) the resulting multilayer biomarker system that spans all preceding biological levels (Section 3.7). Each Results subsection is explicitly linked back to its position within this cascade, and the model is formally depicted in Figure 1 and discussed in an integrated manner in Section 4.3.

## 2. Materials and Methods

### 2.1. Study Design and Rationale

This study was conducted as a narrative review, not a systematic review, to provide a comprehensive and critical synthesis of the current evidence regarding the neuroinflammatory mechanisms and biomarkers linking infectious diseases with psychiatric disorders. Narrative-review methodology was considered the most appropriate approach because the topic encompasses a broad and heterogeneous body of literature, including diverse infectious agents, multiple psychiatric phenotypes, and a wide range of molecular, immunological, neuroimaging, and clinical biomarkers, for which formal quantitative pooling (meta-analysis) is neither feasible nor appropriate. Rather than quantitatively summarizing homogeneous studies, the objective of this review was to integrate evidence from multiple disciplines into a coherent mechanistic framework describing the biological pathways through which infectious diseases may contribute to psychiatric disorders via neuroinflammatory processes.

A structured, transparent, and reproducible search strategy—including defined databases, Boolean search terms, and predefined inclusion/exclusion criteria—was deliberately adopted to strengthen the methodological transparency of this narrative review, consistent with current best-practice recommendations for narrative reviews (SANRA). This structured search process should not be interpreted as indicating a systematic-review methodology: unlike a systematic review, this study does not report a formal risk-of-bias assessment applied uniformly across all included studies, does not perform quantitative/meta-analytic synthesis, and additionally incorporates seminal conceptual publications outside the defined search window via citation tracking (Section 2.3). The review was conducted in accordance with the Scale for the Assessment of Narrative Review Articles (SANRA) recommendations, ensuring methodological transparency, scientific rigor, and critical appraisal appropriate for narrative reviews.

### 2.2. Literature Search Strategy

A comprehensive literature search was performed using four major bibliographic databases to maximize the identification of relevant studies across biomedical, neuroscientific, psychiatric, and translational research disciplines. The following electronic databases were searched: Web of Science Core Collection (Science Citation Index Expanded [SCIE] and Emerging Sources Citation Index [ESCI]); PubMed/MEDLINE; Scopus; and PsycINFO.

The search covered studies published between January 2022 and 30 June 2026. This period was selected to capture the rapid advances in neuroimmunology, particularly the expanding literature concerning NeuroCOVID, HIV-associated neurocognitive disorders, neuroimmune biomarkers, and recent developments in translational psychiatry. Seminal publications published before 2022 were additionally identified through citation tracking and included where they provided essential conceptual or historical background.

Search strategies combined controlled vocabulary (Medical Subject Headings [MeSH] where applicable), database-specific indexing terms, and free-text keywords connected using Boolean operators (AND/OR). The search strategy was adapted to the indexing system and syntax of each database.

The search incorporated four principal conceptual domains:
Domain 1—Biomarkers: (“biomarker*” OR “molecular marker*” OR “inflammatory biomarker*” OR “neuroimaging biomarker*”).
Domain 2—Neuroinflammation: (“neuroinflamm*” OR “neuroinflammation” OR “microglia*” OR “glial activation” OR “astrocyte*” OR cytokine* OR chemokine* OR kynurenine).
Domain 3—Psychiatric Disorders: (“psychiatric disorder*” OR “mental disorder*” OR “mood disorder*” OR “psychotic disorder*” OR depression OR schizophrenia OR “bipolar disorder” OR psychosis OR “anxiety disorder*” OR “cognitive impairment”).
Domain 4—Infectious Diseases: (infection* OR “infectious disease*” OR “viral infection*” OR “bacterial infection*” OR “fungal infection*” OR parasite* OR sepsis OR SARS-CoV-2 OR HIV OR COVID-19).
Boolean operators were used to combine keywords within and between conceptual domains to optimize sensitivity and specificity.

### 2.3. Supplementary Search Strategy

To improve the completeness of the literature search, the reference lists of eligible reviews and key original articles were manually screened (backward citation tracking). Forward citation tracking of highly influential publications was also performed to identify recently published studies relevant to the review objectives.

In addition, seminal publications published before 2022 were incorporated when they substantially contributed to the understanding of neuroinflammatory mechanisms or represented landmark studies within the field.

Grey literature was not systematically searched, consistent with the methodology of narrative reviews. Nevertheless, selected reports issued by international organizations, clinical guidelines, consensus statements, and scientifically relevant preprints were consulted where appropriate to provide epidemiological context, methodological clarification, or emerging evidence not yet extensively represented in the peer-reviewed literature.

### 2.4. Study Selection

Study selection was performed in two consecutive stages.

During the first stage, all retrieved records were screened based on titles and abstracts to determine their relevance to the review topic. Studies investigating neuroinflammatory mechanisms, neuroimmune interactions, inflammatory biomarkers, infectious diseases, and psychiatric disorders were considered potentially eligible.

During the second stage, the full texts of potentially relevant publications were evaluated according to predefined inclusion and exclusion criteria.

Inclusion criteria comprised: peer-reviewed original research articles; systematic reviews and meta-analyses; narrative reviews; studies published in English; studies published between January 2022 and 30 June 2026, with seminal earlier publications included through citation tracking; and studies investigating neuroinflammatory mechanisms, biomarkers, or neuropsychiatric manifestations associated with infectious diseases.

Exclusion criteria comprised: conference abstracts; editorials; letters; commentaries; brief reviews lacking substantial scientific synthesis; duplicate publications; studies unrelated to infection-associated neuroinflammation or psychiatric disorders; and studies exclusively addressing non-infectious neuroinflammatory conditions without discussing infectious mechanisms.

Disagreements regarding study eligibility were resolved through discussion and consensus.

### 2.5. Data Extraction and Narrative Synthesis

Because of the substantial heterogeneity in the included literature, a standardized data extraction process was used to collect the most relevant information from each publication.

For mechanistic and experimental studies, extracted information included the infectious agent investigated, neuroimmune pathways, inflammatory mediators, biomarkers, the experimental model, and principal mechanistic findings.

For clinical studies, extracted information included study population characteristics, the infectious disease investigated, psychiatric outcomes, biomarker assessment methods, and principal clinical findings.

For systematic reviews, meta-analyses, and narrative reviews, the review scope, principal conclusions, methodological strengths, and identified knowledge gaps were recorded.

The evidence was synthesized narratively and organized according to the thematic structure of the Unified Neuroimmune Framework introduced in Section 1, integrating findings related to systemic inflammation, neuroinflammation, gut microbiota, blood–brain barrier dysfunction, central nervous system infections, neuropsychiatric manifestations, immune-mediated psychiatric disorders, immune dysfunction, and neuroinflammatory biomarkers.

Because of the diversity of study designs, populations, biomarkers, and clinical outcomes, quantitative synthesis or meta-analysis was not considered appropriate.

### 2.6. Critical Appraisal

Considering the broad range of study designs included, methodological quality was critically evaluated according to study type.

Systematic reviews and meta-analyses were evaluated with particular attention to methodological rigor, literature search strategy, risk-of-bias assessment, and consistency of findings.

Original research articles were critically appraised according to study design, sample size, participant selection, biomarker methodology, statistical analyses, control of confounding factors, and reproducibility.

Narrative reviews were assessed regarding comprehensiveness, scientific balance, transparency of methodology, integration of current evidence, and identification of existing knowledge gaps.

Rather than formally excluding studies based solely on methodological limitations, the quality of the available evidence was considered throughout the interpretation and synthesis of findings, and specific methodological caveats (e.g., small sample sizes, cross-sectional design, or lack of replication) are flagged explicitly at relevant points in Section 3 and summarized comparatively in Section 4.6.

### 2.7. Search Outcomes

Study selection followed a structured narrative-review approach. A total of 561 records were initially identified through database searching. After duplicate removal, 387 unique records remained for title and abstract screening, of which 215 were excluded because they did not meet the predefined eligibility criteria. The full texts of the remaining 172 articles were assessed for eligibility, resulting in the inclusion of 76 studies in the final qualitative synthesis, comprising 42 original research articles, 22 systematic reviews and meta-analyses, and 12 narrative reviews or expert consensus papers. In addition, 12 seminal publications published before 2022 were identified through citation tracking and incorporated to provide historical and conceptual context. The overall selection process is illustrated in Figure 1, which summarizes the identification, screening, eligibility assessment, and final inclusion of the literature analyzed in this narrative review.

This diagram is presented as a narrative-review study-selection flow diagram (not a PRISMA flow diagram), consistent with the SANRA-based methodology described in Section 2.1.

## 3. Results

Inflammation is a fundamental biological process involved in protecting the host against infectious agents and tissue injury. Acute inflammation plays an adaptive and beneficial role. However, persistent or dysregulated inflammatory responses may contribute to pathological mechanisms involved in aging and the development of numerous chronic diseases, including psychiatric disorders [12].

In recent years, advances in neuroimmunology have demonstrated that infections can initiate or sustain both systemic and central inflammatory responses, thereby influencing nervous system function through the immune–brain axis. Infection-induced inflammation may be further amplified by additional pro-inflammatory factors, including obesity and metabolic dysfunction, gut dysbiosis, psychological stress, and sleep disturbances [8].

### 3.1. Infection, Systemic Inflammation, and Neuroinflammation

Infectious agents (viral, bacterial, fungal, or parasitic) express pathogen-associated molecular patterns (PAMPs), which are recognized by pattern recognition receptors (PRRs) of the innate immune system. Activation of PRRs triggers multiple pro-inflammatory signaling cascades, including NF-κB, MAPK, and PI3K/Akt, as well as the production of reactive oxygen and nitrogen species [13,14,15].

Activation of the transcription factor NF-κB induces the expression of genes involved in the inflammatory response, including TNF-α, IL-6, and the precursor pro-IL-1β. In parallel, activation of the NLRP3 inflammasome promotes the proteolytic maturation of pro-IL-1β and pro-IL-18, thereby amplifying the inflammatory response. This process is subsequently sustained by damage-associated molecular patterns (DAMPs) released from injured tissues, which reactivate PRRs and contribute to the maintenance of inflammation [16,17,18,19].

Under conditions of persistent inflammatory stimulation, the immune response evolves toward a sustained pro-inflammatory cytokine profile characterized by continued expression of TNF-α, IL-1β, and IL-6, together with IFN-γ, IL-17, TGF-β, and chemokines involved in the continuous recruitment of immune cells, tissue remodeling, and fibrosis [20].

Systemic inflammation driven by pro-inflammatory cytokines activates indoleamine 2,3-dioxygenase (IDO), diverting tryptophan metabolism toward the kynurenine pathway and reduce serotonin synthesis. Concurrently, this pathway generates neuroactive metabolites, including quinolinic acid, an agonist of N-methyl-D-aspartate (NMDA) receptors, which contributes to glutamate-mediated excitotoxicity and synaptic dysfunction [21,22,23].

### 3.2. Infection, Gut Microbiota, and the Blood–Brain Barrier

In addition to inducing systemic inflammatory responses, infections and exposure to antibiotics can disrupt the composition and function of the gut microbiota, promoting gut dysbiosis. Dysbiosis alters the expression of tight-junction (TJ) proteins, including zonula occludens-1 (ZO-1), occludin, and claudins, thereby increasing intestinal permeability and facilitating the translocation of microbial products, particularly lipopolysaccharide (LPS), into circulation. This process further amplifies innate immune activation and contributes to the maintenance of systemic inflammation [24].

Subsequently, circulating inflammatory mediators compromise the integrity of the blood–brain barrier (BBB) through mechanisms involving endothelial activation, oxidative stress, and disruption of endothelial tight-junction proteins, resulting in increased BBB permeability. This facilitates the passage of circulating cytokines, immune cells, and microbial products into the central nervous system, thereby promoting neuroimmune communication and creating conditions that favor the development and persistence of neuroinflammatory responses [25]. BBB dysfunction, together with sustained exposure to circulating inflammatory mediators, promotes the activation of microglia and astrocytes, leading to the release of pro-inflammatory cytokines, reactive oxygen and nitrogen species, and other neurotoxic mediators. During persistent immune activation, chronic microglial activation, excessive cytokine production, and infiltration of peripheral T lymphocytes disrupt neurotransmission, impair neuroplasticity, and alter neuronal network function. These processes contribute to aberrant synaptic pruning, neuronal dysfunction, and progressive neurodegeneration, ultimately increasing vulnerability to infection-associated neuropsychiatric disorders [15,26].

### 3.3. Central Nervous System Infections and Neuroinflammation

Infectious agents can cause direct injury to the central nervous system (CNS) through hematogenous dissemination, retrograde neuronal transport, or direct inoculation. Invasion of neural tissue results in neuronal and glial injury associated with neuroinflammation, which may be further amplified by secondary mechanisms, including vascular injury, cerebral edema, mass effect, and increased intracranial pressure [12].

These processes lead to microvascular dysfunction, hypoperfusion, and tissue ischemia, resulting in the release of DAMPs and the activation of PRRs within the CNS [8]. In this context, cerebral edema and elevated intracranial pressure are not merely consequences of CNS infection but also act as amplifiers of the neuroinflammatory response, perpetuating tissue injury and immune activation [15].

### 3.4. Neuropsychiatric Manifestations Associated with Infections

Neuroinflammation is associated with a heterogeneous spectrum of neuropsychiatric manifestations and biomarker profiles. The phenotypic expression of neuroinflammation depends on the infectious agent involved and its tissue tropism, as well as the magnitude and duration of the host immune response, resulting in considerable variability in both clinical presentation and molecular signatures. Table 1 presents selected representative infectious disease models, including systemic infection (sepsis) and bacterial, viral, and parasitic infections, chosen to illustrate the diversity of pathophysiological mechanisms, neurobiological targets, and neuropsychiatric phenotypes. The examples were selected based on their clinical relevance and the strength of available evidence and are intended to be illustrative rather than exhaustive.

Among the various infectious diseases associated with neuropsychiatric manifestations, Neuro-COVID and HIV-associated neurocognitive disorders (HAND) represent two paradigmatic clinical models of infection-associated neuroinflammation. Although both conditions share common neuroimmune mechanisms, including cytokine-mediated inflammation, microglial activation, and blood–brain barrier dysfunction, they differ in their predominant pathogenic drivers.

#### 3.4.1. Features of Neuroinflammation in COVID-19

The association between neuroinflammation and psychiatric manifestations in COVID-19 is reflected in a characteristic affective–cognitive phenotype, in which depression, anxiety, sleep disturbances, and persistent cognitive impairment emerge as manifestations of integrated neuroimmune dysfunction. During the acute phase of COVID-19, neuropsychiatric manifestations are primarily driven by systemic inflammation, innate immune activation, endothelial dysfunction, and BBB disruption, whereas long COVID is characterized by persistent low-grade neuroinflammation and neurovascular dysfunction that are thought to sustain affective and cognitive symptoms [27,28]. This phenotype is particularly evident in long COVID, where sustained inflammation is considered a major contributor to symptom persistence [29]. Activation of innate immune pathways and cytokine signaling is thought to modulate cortico-limbic circuits involved in emotion and cognition, although considerable interindividual variability exists [30,31]. Persistent cognitive impairment has also been associated with astroglia injury and alterations in cerebral perfusion consistent with immune–vascular coupling, supporting the contribution of ongoing neuroimmune and neurovascular dysfunction to long COVID [32,33,34]. Although several inflammatory and neuroglial biomarkers have been investigated, no single biomarker has demonstrated sufficient predictive accuracy for long-term neuropsychiatric outcomes [35,36]. Experimental models further support the concept that sustained immune dysregulation, rather than direct viral neuroinvasion, is the principal driver of persistent neuropsychiatric sequelae [37]. Collectively, these findings support inflammation as a transdiagnostic mechanism underlying post-COVID neuropsychiatric manifestations and increased vulnerability to affective and behavioral disorders [38,39].

#### 3.4.2. Features of Neuroinflammation in HIV

Neuroinflammation is a central pathological feature of HIV infection and remains detectable despite sustained viral suppression achieved through combination antiretroviral therapy (ART). Although the incidence of severe HIV-associated dementia has markedly declined in the ART era, milder forms of HIV-associated neurocognitive disorders (HAND) continue to affect a substantial proportion of people living with HIV. Current evidence indicates that persistent immune activation within both the CNS and the periphery contributes to ongoing neuronal dysfunction, cognitive impairment, and neuropsychiatric manifestations, including depression, anxiety, and apathy. Rather than representing a direct consequence of uncontrolled viral replication, contemporary HAND is increasingly viewed as the result of chronic low-grade neuroinflammation interacting with aging, vascular risk factors, metabolic comorbidities, and other host-related determinants. Recent updates from the International HIV-Cognition Working Group further emphasize that cognitive impairment in treated HIV infection represents a heterogeneous clinical spectrum requiring multidimensional assessment beyond the traditional HAND classification [40].

A key mechanism underlying persistent neuroinflammation involves dysregulated communication between immune and neural cells. Extracellular vesicles released from HIV-infected macrophages, microglia, astrocytes, and other cell populations transport inflammatory cytokines, viral proteins, and regulatory microRNAs that facilitate the propagation of inflammatory signaling throughout the CNS [41]. This altered intercellular communication contributes to sustained microglial activation, astrocytic dysfunction, synaptic injury, and progressive neuronal damage, thereby promoting cognitive decline even in the absence of detectable viral replication within the cerebrospinal fluid [42]. In parallel, systemic immune activation remains highly heterogeneous among individuals living with HIV and is influenced by host-specific factors, including biological sex, age, genetic background, and immune status, all of which contribute to interindividual variability in neurological outcomes [43].

From a clinical perspective, persistent central and peripheral inflammation plays a major role in shaping the neuropsychiatric phenotype of HIV infection. Chronic immune activation has been associated with impairments in attention, executive function, information-processing speed, and memory, while simultaneously contributing to depressive symptoms and emotional dysregulation through disruption of the immune–brain axis [10]. Peripheral inflammatory biomarkers are increasingly being investigated as indicators of ongoing neuroinflammatory activity and disease progression, particularly in patients with concomitant systemic comorbidities. For example, methamphetamine use has been shown to potentiate systemic immune activation, exacerbate neuroinflammatory pathways, and accelerate biological dysfunction associated with HIV infection, thereby increasing the risk of neurocognitive deterioration [44].

Overall, neuroinflammation in Neuro-AIDS is sustained by persistent immune activation, dysregulated intercellular communication, chronic glial activation, and impaired neuroimmune homeostasis. These interconnected mechanisms contribute to ongoing cognitive impairment and neuropsychiatric symptoms despite effective viral suppression. Accordingly, current perspectives advocate a multidimensional approach to the evaluation of cognitive dysfunction in people living with HIV, integrating neuroinflammatory mechanisms with aging, comorbidities, and psychosocial factors [40].

### 3.5. Psychiatric Disorders as Immune-Mediated Phenotypes

Accumulating evidence supports the concept that psychiatric disorders represent immune-mediated phenotypes, characterized by complex interactions among systemic inflammation, neuroinflammation, and dysfunction of neuronal networks involved in emotional regulation, motivation, and cognition. Within this framework, inflammation is not merely an epiphenomenon of disease but a biological mechanism capable of influencing clinical presentation, symptom severity, and treatment response [45].

Persistent activation of the innate immune system, triggered by infections, chronic stress, or other pro-inflammatory stimuli, promotes the release of pro-inflammatory cytokines and reactive oxygen species, leading to oxidative stress and altered neurotransmission. These processes reduce dopaminergic neurotransmission while enhancing glutamatergic signaling through excitotoxic mechanisms, thereby contributing to anhedonia, fatigue, depressive symptoms, and cognitive impairment. Consequently, inflammation exerts transdiagnostic effects, contributing not only to affective disorders but also to schizophrenia and other psychotic disorders [45].

Concurrently, activation of monocytes and T lymphocytes is accompanied by metabolic reprogramming and enhanced communication between the peripheral immune system and the central nervous system, facilitating immune cell trafficking and amplification of the neuroinflammatory response. Cytokines produced both peripherally and within the CNS modulate neuronal function through their effects on neurotransmitter systems and the structural integrity of white matter, thereby contributing to the remodeling of brain circuits implicated in psychopathology [46].

### 3.6. Neurotrophic Factors as a Downstream Mediator of Neuroinflammation

**Figure 2:**
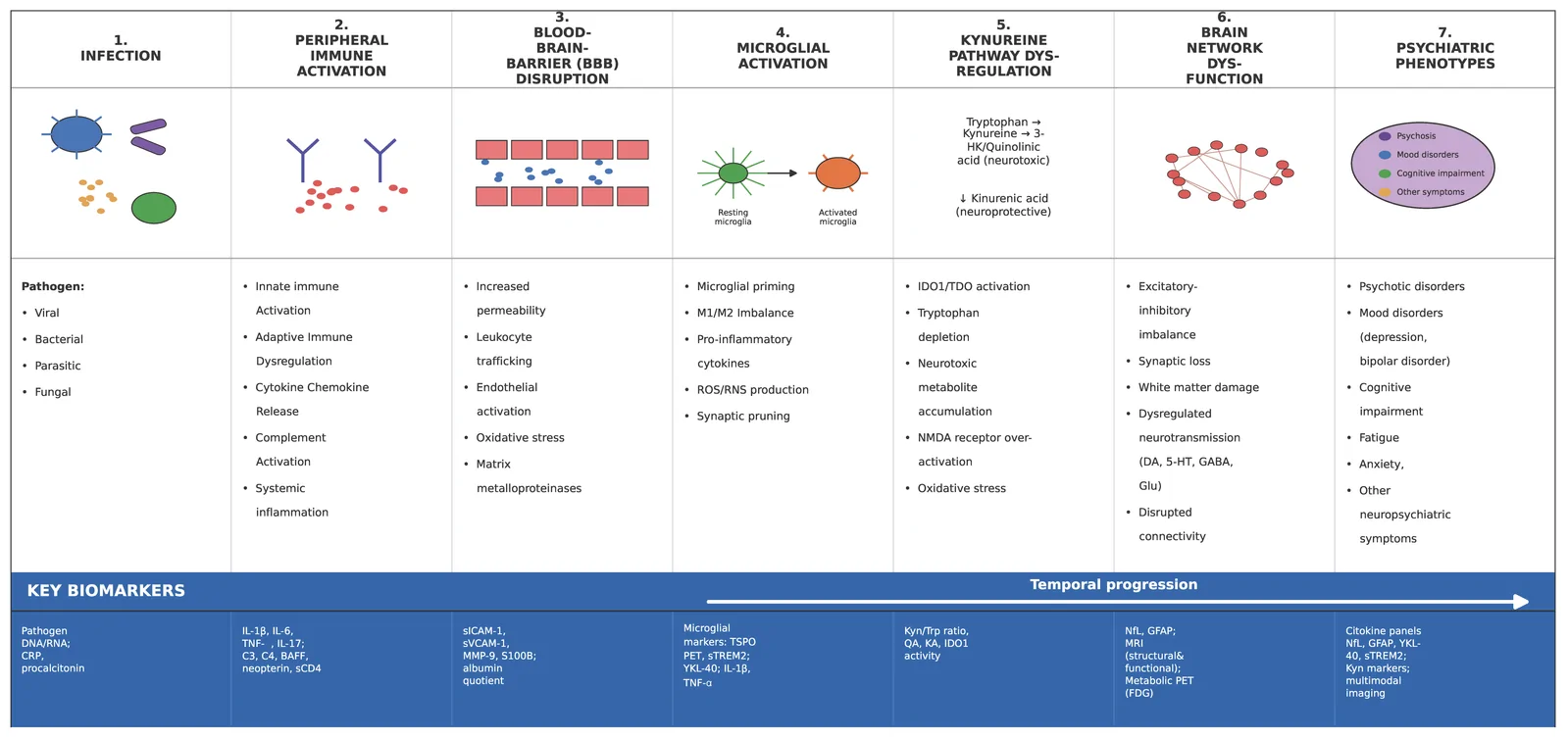
Proposed unified neuroimmune framework linking infections to neuropsychiatric disorders. Legend: BBB = blood–brain barrier; BAFF = B-cell-activating factor; BDNF = brain-derived neurotrophic factor; CRP = C-reactive protein; CSF = cerebrospinal fluid; DA = dopamine; FDG = fluorodeoxyglucose; GFAP = glial fibrillary acidic protein; Glu = glutamate; HPA = hypothalamic–pituitary–adrenal; IDO = indoleamine 2,3-dioxygenase; IFN-γ = interferon gamma; IL = interleukin; Kyn = kynurenine; KA = kynurenic acid; MIP = macrophage inflammatory protein; MMP = matrix metalloproteinase; MRI = magnetic resonance imaging; NfL = neurofilament light chain; NGF = nerve growth factor; PET = positron emission tomography; QA = quinolinic acid; ROS = reactive oxygen species; sTREM2 = soluble triggering receptor expressed on myeloid cells 2; TNF-α = tumor necrosis factor alpha; Trp = tryptophan; TSPO = translocator protein.

A growing body of evidence indicates that chronic exposure to pro-inflammatory cytokines, particularly IL-1β and TNF-α, suppresses the expression and signaling of brain-derived neurotrophic factors (BDNF), a key regulator of synaptic plasticity, neurogenesis, and neuronal survival. Reduced BDNF availability has been consistently associated with impaired hippocampal neuroplasticity and depressive phenotypes, suggesting that neurotrophic suppression represents a mechanistic link between peripheral/central inflammation and the affective and cognitive symptoms described above [47,48]. In parallel, nerve growth factor (NGF)-responsive elements have been shown to modulate immune-cell inflammatory activity and are themselves dysregulated in neuroinflammatory disease models, indicating a bidirectional relationship between neurotrophic and immune signaling rather than a purely unidirectional cytokine-to-neurotrophin suppression [49]. Pharmacological and clinical evidence further suggests that restoration of neurotrophic signaling may partly underline the therapeutic benefit of some anti-inflammatory and antidepressant interventions [50,51]. Neurotrophic dysregulation is therefore incorporated into the Unified Neuroimmune Framework as a distinct downstream biological axis linking sustained inflammation to structural and functional neuroplastic impairment (Table 2, Figure 2).

Recent neuroimaging studies demonstrate that inflammation is associated with both functional and structural dysconnectivity within brain circuits relevant to transdiagnostic psychiatric symptoms. The most consistent alterations have been reported in frontostriatal circuits involved in reward processing and motivation, amygdala–prefrontal circuits responsible for fear and anxiety processing, frontolimbic networks involved in emotional regulation and interoception, and somatomotor networks associated with psychomotor slowing. Reduced functional connectivity within these networks correlates with circulating inflammatory markers, including interleukin-6 (IL-6), tumor necrosis factor-α (TNF-α), interferons (IFNs), and C-reactive protein (CRP), suggesting the existence of a common neurobiological substrate underlying symptoms such as anhedonia, anxiety, and psychomotor retardation [46,52].

The clinical relevance of these observations is further supported by pharmacological studies demonstrating that restoration of dopaminergic neurotransmission can normalize functional connectivity in patients with elevated inflammatory burden. For example, levodopa administration has been shown to increase functional connectivity within the ventromedial prefrontal cortex and improve anhedonia in patients with depression and elevated CRP concentrations, suggesting that resting-state functional connectivity (rsFC) may represent a modifiable neuroimaging biomarker of inflammation-related brain dysfunction [46].

Collectively, these findings support the reconceptualization of psychiatric disorders as immune-mediated phenotypes, in which immune dysregulation, altered neurotransmission, impaired neurotrophic support, and remodeling of brain circuits converge to produce a common spectrum of clinical manifestations. This perspective provides a rationale for the identification of multimodal biomarkers and the development of personalized therapeutic strategies targeting patient subgroups characterized by distinct inflammatory profiles [45,52].

### 3.7. Pathophysiological Mechanisms of Immune Dysfunction in Psychiatric Disorders

The persistence of immune dysfunction in psychiatric disorders is sustained by pathophysiological mechanisms that extend beyond neuroinflammation itself and involve complex interactions among the neuroendocrine, autonomic, and immune systems. Chronic hyperactivation of the hypothalamic–pituitary–adrenal (HPA) axis leads to glucocorticoid resistance, thereby reducing the anti-inflammatory effects of cortisol [53]. Concurrently, sympathetic predominance and reduced vagal tone impair the cholinergic anti-inflammatory reflex, promoting persistent immune activation [53,54]. This response is further amplified by low-grade systemic inflammation, maintained in part through disruption of the intestinal barrier described in Section 3.2 and translocation of bacterial lipopolysaccharide (LPS) [55]. Prolonged exposure to these mechanisms ultimately results in immunosenescence, characterized by telomere shortening, increased apoptosis of immune cells, and impaired immune competence [54]. Collectively, these processes explain why patients with psychiatric disorders exhibit not only neuroinflammation but also increased susceptibility to infections, inflammatory comorbidities, and dysregulated immune responses [54,55].

These pathophysiological alterations have important clinical implications. Large population-based cohort studies have demonstrated that individuals with psychiatric disorders are at significantly increased risk of developing severe infections and infection-related complications, even after adjustment for demographic, lifestyle, and medical confounders [56]. Similarly, systematic reviews and meta-analyses have shown that patients with schizophrenia and bipolar disorder experience a higher incidence of severe infections and increased infection-related mortality, highlighting immune dysfunction as a shared biological mechanism underlying both psychiatric illness and susceptibility to infectious diseases [3,57].

The neurobiological consequences of infection-induced immune activation extend beyond postnatal life, with increasing evidence indicating that prenatal inflammatory exposures can shape brain development and confer long-term vulnerability to neuropsychiatric disorders [58]. Prenatal exposure to certain infectious agents has been associated with an increased risk of neurodevelopmental and psychiatric disorders, including schizophrenia (SCZ), bipolar disorder (BD), and autism spectrum disorder (ASD) [59]. In most cases, infectious pathogens do not directly cross the placenta; rather, maternal immune activation (MIA) induces the production of inflammatory cytokines that alter placental function and interfere with fetal brain development [59]. During pregnancy, fetal development depends on a tightly regulated balance between pro-inflammatory and anti-inflammatory cytokines. Maternal infections may disrupt this immune equilibrium, resulting in placental dysfunction, altered fetal neurodevelopment, and long-lasting changes in fetal immune programming [59]. Although maternal pathogen-specific antibodies readily cross the placenta and may serve as markers of maternal infection, current evidence suggests that they are not the primary mediators of the neurodevelopmental effects associated with maternal immune activation [60,61].

### 3.8. Neuroinflammatory Biomarkers and Psychiatric Phenotypes

Neuroinflammatory biomarkers provide an integrated framework for understanding how infection-induced immune responses contribute to the development and persistence of psychiatric disorders. Rather than representing isolated biological indicators, these biomarkers reflect interconnected processes involving innate and adaptive immunity, central neuroimmune activation, metabolic and epigenetic regulation, and neurotrophic signaling, as well as autonomic and neuroendocrine dysfunction. Current evidence therefore supports a multidimensional biomarker approach that integrates peripheral and central measures to improve the biological characterization of infection-associated psychiatric phenotypes [62]. A comprehensive synthesis of these biomarker axes is provided in Table 2, which summarizes their biological domains, representative markers, associated infections (specified as infection-specific or general), and associated psychiatric phenotypes.

Advances in molecular profiling and neuroimaging have substantially expanded the range of candidate biomarkers. Peripheral inflammatory mediators capture systemic immune activation, whereas imaging techniques provide in vivo evidence of neuroimmune dysfunction and altered brain network organization. Emerging data further indicate that persistent inflammatory signaling may induce long-lasting alterations in synaptic plasticity and memory-related pathways, while disturbances of the sleep–immune axis may amplify immune dysregulation and increase vulnerability to psychiatric symptoms [63,64]. Among neuroimaging modalities, TSPO-PET has become the most extensively investigated marker of microglial activation, although methodological heterogeneity and limited cellular specificity continue to restrict its clinical applicability [65]. Complementary multimodal imaging approaches further support the association between neuroinflammation and structural and functional alterations in brain networks involved in cognition and emotional regulation [66].

Recent advances have also highlighted biomarkers reflecting glial activation and neuroaxonal injury. Glial fibrillary acidic protein (GFAP) serves as a marker of astrocytic activation and blood–brain barrier dysfunction, whereas neurofilament light chain (NfL) is considered one of the most sensitive indicators of neuroaxonal damage. YKL-40 has emerged as a promising biomarker of persistent neuroinflammation, particularly in central nervous system infections, while soluble triggering receptors expressed on myeloid cells 2 (sTREM2) is increasingly investigated as a marker of microglial activation. Together, these biomarkers complement cytokine-based and imaging biomarkers by providing additional information on astrocytic dysfunction, neuronal injury, and innate immune activation, thereby contributing to a more comprehensive characterization of infection-associated neuropsychiatric disorders [67,68].

Despite considerable progress, no single biomarker has demonstrated sufficient sensitivity or specificity for routine clinical use. Instead, current evidence favors integrated biomarker panels combining immunological, molecular, imaging, and physiological measures, although further methodological standardization, external validation, and longitudinal studies remain necessary before these approaches can be incorporated into clinical practice [69].

**Table 2:** Multilayer predictive biomarker system for infection-associated psychiatric disorders

Biological Axis	Main Biomarkers	Assoc. Infections (Specificity)	Mechanism	Predictive Phenotype	Ref.
Inflammatory axis (innate immunity)	IL-6, TNF-α, IL-1β, MCP-2, MIP-1β, CRP	General—SARS-CoV-2, HIV, respiratory infections, sepsis	NF-κB activation; systemic inflammatory response; peripheral–CNS signaling	Depression, anxiety, psychosis	[53,54,55,56,57,58,59,60,61,62,63,64,65,66,67,68,69,70]
Adaptive immunity & autoimmunity axis	BAFF, C4, T/B-cell subsets, HERVs	Infection-specific—HSV, EBV, HIV, chronic viral infections	B-cell activation; complement-mediated synaptic pruning; endogenous retroviral activation	Schizophrenia, bipolar disorder, psychosis	[69,71,72,73]
Central neuroimmune axis (imaging)	TSPO PET, rs-fMRI, DTI, FDG-PET	General—HIV, SARS-CoV-2, viral encephalitis	Microglial activation; frontolimbic dysconnectivity; hypometabolism	Depression, schizophrenia, anhedonia	[46]
Metabolomic axis	Kynurenine, quinolinic acid, TRP/KYN ratio	General—viral and bacterial infections	IDO activation; glutamatergic excitotoxicity	Depression, psychosis, cognitive fatigue	[74]
Neurotrophic axis (NEW)	BDNF, NGF	General—viral, bacterial, post-infectious inflammatory states	Cytokine-mediated suppression of neurotrophic signaling; impaired synaptic plasticity/neurogenesis	Depression, cognitive impairment, poor treatment response	[47,48,49,50,51]
Epigenetic & transcriptomic axis	miR-146a, miR-155, DNA methylation patterns	General—chronic infections, post-viral syndromes	Persistent immune reprogramming; inflammaging; HPA-axis regulation	Chronic depression, relapse vulnerability	[56,72]
Autonomic & neuroendocrine axis	Cortisol, ACTH, HRV, vagal tone	General—sepsis, systemic infections, post-viral states	HPA-axis hyperactivation; glucocorticoid resistance; reduced vagal tone	Anxiety, PTSD, somatic depression	[53,55]
Gut–brain axis & immunosenescence	LPS, zonulin, SCFA, telomere length, CD28− T cells	General—chronic bacterial infections, dysbiosis	LPS translocation; TLR4 activation; immunosenescence	Persistent depression, cognitive decline	[12]

Legend: BAFF = B-cell-activating factor; BDNF = brain-derived neurotrophic factor; C4 = complement component 4; CRP = C-reactive protein; DTI = diffusion tensor imaging; EBV = Epstein–Barr virus; FDG-PET = fluorodeoxyglucose positron emission tomography; HERVs = human endogenous retroviruses; HPA axis = hypothalamic–pituitary–adrenal axis; HRV = heart rate variability; HSV = herpes simplex virus; IDO = indoleamine 2,3-dioxygenase; IL = interleukin; LPS = lipopolysaccharide; MCP-2 = monocyte chemoattractant protein-2; MIP-1β = macrophage inflammatory protein-1 beta; NF-κB = nuclear factor kappa-light-chain-enhancer of activated B cells; NGF = nerve growth factor; PET = positron emission tomography; PTSD = post-traumatic stress disorder; rs-fMRI = resting-state functional magnetic resonance imaging; SCFA = short-chain fatty acid; SARS-CoV-2 = severe acute respiratory syndrome coronavirus 2; T/B cells = T lymphocytes/B lymphocytes; TNF-α = tumor necrosis factor alpha; TRP/KYN ratio = tryptophan/kynurenine ratio; TSPO = translocator protein; TLR4 = Toll-like receptor 4.

## 4. Discussion

### 4.1. Neuroinflammation as the Biological Link Between Infection and Psychiatric Disorders

The evidence synthesized in this review supports neuroinflammation as a shared biological mechanism linking diverse infectious diseases to a broad spectrum of psychiatric manifestations. Despite marked differences in pathogen biology, infectious agents converge on common neuroimmune pathways characterized by persistent peripheral immune activation, cytokine signaling, microglial dysfunction, metabolic dysregulation, impaired neurotrophic support, and altered brain connectivity. This convergence provides a mechanistic framework for understanding why depression, anxiety, psychosis, cognitive impairment, fatigue, and other neuropsychiatric manifestations occur across clinically distinct infectious conditions. Rather than representing isolated disease-specific phenomena, these manifestations appear to reflect common biological responses driven by sustained neuroimmune dysregulation, as formalized in the Unified Neuroimmune Framework introduced in Section 1 and applied throughout Section 3.

### 4.2. Biomarkers as Translational Tools

The expanding repertoire of neuroinflammatory biomarkers has substantially strengthened the understanding of the relationship between infection, neuroinflammation, and psychiatric disorders. Peripheral inflammatory mediators, markers of adaptive immune activation, molecular signatures, neurotrophic markers, and advanced neuroimaging techniques provide complementary evidence that infection-induced immune responses extend beyond the acute phase and are accompanied by persistent alterations in central nervous system function. Consequently, neuroinflammatory biomarkers have become valuable translational tools for investigating disease mechanisms, identifying biologically distinct patient subgroups, and monitoring neuroimmune activity. However, no individual biomarker currently demonstrates sufficient diagnostic accuracy or specificity for routine clinical use. Future diagnostic approaches will likely rely on multimodal biomarker panels integrating immunological, molecular, metabolic, and neuroimaging measures to improve biological stratification and support precision psychiatry [65,66,67].

### 4.3. Integrated Neuroimmune Model

Collectively, the findings support the Unified Neuroimmune Framework outlined in Section 1, in which infection initiates a dynamic cascade involving peripheral immune activation, adaptive immune dysregulation, metabolic reprogramming, neurotrophic suppression, neuroendocrine alterations, and changes in functional brain networks. Rather than acting as isolated processes, these mechanisms interact across multiple biological levels, reinforcing persistent neuroinflammation and increasing susceptibility to transdiagnostic psychiatric phenotypes. Emerging evidence further suggests that disturbances of the sleep–immune axis, together with long-lasting molecular and epigenetic adaptations, may contribute to the persistence of psychiatric symptoms long after resolution of the acute infection [53,63,64]. Complementary molecular–electrophysiological approaches, such as dopamine transporter imaging combined with electroencephalographic markers, have likewise shown value for characterizing neurodegenerative–neuroinflammatory overlap syndromes, as illustrated by recent findings in dementia with Lewy bodies [75]. Emerging frameworks emphasizing bidirectional crosstalk between systemic metabolism and neuroimmune signaling—termed neuroimmunometabolism—further underscore the importance of incorporating metabolic regulators into future multidimensional biomarker panels [76].

Figure 2 summarizes the proposed unified framework linking infection to neuropsychiatric disorders. Following infection, peripheral immune activation promotes systemic inflammation and blood–brain barrier dysfunction, facilitating communication between the peripheral immune system and the central nervous system. Subsequent microglial activation and dysregulation of the kynurenine pathway contribute to neurotoxic metabolite accumulation, synaptic dysfunction, and large-scale brain network alterations, while concurrent suppression of neurotrophic signaling (BDNF, NGF) further impairs neuroplasticity, ultimately leading to heterogeneous psychiatric phenotypes. The model also highlights representative biomarkers associated with each biological stage, emphasizing that no single biomarker adequately captures the complexity of infection-associated neuropsychiatric disorders and supporting the use of multimodal biomarker panels.

### 4.4. Clinical Implications

From a clinical perspective, these findings emphasize the need for an integrated multidisciplinary approach to infection-associated psychiatric disorders. Early recognition of neuropsychiatric symptoms following infection, combined with comprehensive assessment of infectious, neurological, psychiatric, and immunological factors, may facilitate timely intervention and improve long-term outcomes. Although current management remains primarily symptom-oriented, individualized treatment strategies addressing both the underlying infectious disease, when appropriate, and psychiatric manifestations should be complemented by psychosocial interventions and longitudinal follow-up.

Toward Mechanism-Based Pharmacological Strategies: Several repurposed anti-inflammatory and immunomodulatory agents are currently under clinical investigation as adjunctive treatments for infection-associated and inflammation-related psychiatric conditions, illustrating how the biomarker axes described above may translate into practical clinical psychiatry. Minocycline, a tetracycline with microglia-inhibiting and anti-inflammatory properties independent of its antimicrobial action, has been studied as adjunctive therapy for depressive and post-infectious cognitive symptoms associated with elevated inflammatory markers. Tocilizumab, a monoclonal antibody targeting the IL-6 receptor, has been investigated in inflammation-associated depressive phenotypes characterized by elevated peripheral IL-6, directly reflecting the inflammatory axis summarized in Table 2. COX-2 inhibitors (e.g., celecoxib) have similarly been evaluated as adjuncts to conventional antidepressant therapy in patients with biomarker evidence of low-grade systemic inflammation. While none of these agents is currently approved for primary psychiatric indications, their investigation illustrates the translational potential of the biomarker-guided, mechanism-based approach proposed in this review, and supports the rationale for future trials stratifying patients by inflammatory and neurotrophic biomarker profile rather than by psychiatric diagnosis alone.

A better understanding of neuroinflammatory mechanisms may ultimately facilitate biological patient stratification and support the development of more personalized preventive and therapeutic approaches.

### 4.5. Future Research Perspectives

Future research should move beyond the investigation of isolated inflammatory markers toward integrated neuroimmune models capable of capturing the dynamic interactions between peripheral immune activation, central neuroinflammation, metabolic dysfunction, neurotrophic signaling, and alterations in brain connectivity. Longitudinal multimodal studies incorporating serial biomarker assessment, advanced neuroimaging, and multi-omics technologies will be essential to characterize the temporal evolution of neuroinflammatory processes and identify biologically distinct endophenotypes of infection-associated psychiatric disorders. Standardization of biomarker acquisition and analytical methodologies, together with validation in independent cohorts, will be critical for improving reproducibility and facilitating clinical translation. In parallel, interventional studies targeting neuroinflammatory pathways, including the repurposed agents discussed in Section 4.4—are needed to determine whether modulation of immune responses can prevent or attenuate psychiatric manifestations following infectious diseases, thereby establishing neuroinflammation not only as a mechanistic link but also as a clinically actionable therapeutic target.

### 4.6. Limitations

Several limitations should be acknowledged when interpreting the findings of this review. First, as a narrative review employing a structured but non-systematic search and selection process, this study is inherently susceptible to selection and interpretative bias because it does not employ a formal, uniformly applied risk-of-bias assessment or the quantitative synthesis procedures required for a systematic review or meta-analysis. Consequently, the proposed framework should be interpreted as a conceptual and mechanistic integration of the available evidence rather than as evidence capable of quantifying effect sizes or establishing causal relationships between infectious diseases, neuroinflammation, and psychiatric disorders.

Second, the substantial heterogeneity in the included studies with respect to infectious agents, patient populations, biomarker methodologies, neuroimaging techniques, and psychiatric outcomes limits direct comparisons across studies and, as noted at several points in Section 3, likely contributes to inconsistent findings across cohorts—for example, the divergent predictive performance of inflammatory biomarkers reported in post-viral psychiatric sequelae studies (Section 3.4.1). Third, the predominance of cross-sectional and observational study designs within the available literature precludes firm conclusions regarding the temporal sequence and causality of the observed neuroimmune mechanisms. Furthermore, the incomplete integration of longitudinal clinical data, multi-omics approaches, and advanced neuroimaging currently limits the robustness and external validity of predictive neuroimmune models. Finally, methodological variability, differences in biomarker acquisition and analytical procedures, and the lack of standardized validation protocols remain major barriers to the translation of neuroinflammatory biomarkers into routine clinical practice.

Despite these limitations, the present review provides an integrative and biologically grounded framework that synthesizes current evidence across multiple infectious diseases, neuroimmune pathways, and psychiatric phenotypes, while identifying key knowledge gaps and priorities for future translational research.

## 5. Conclusions

This review supports neuroinflammation as a unifying framework linking infectious diseases with a broad spectrum of psychiatric disorders through interconnected immune, metabolic, neurotrophic, vascular, and neural mechanisms. The integration of current evidence into a single conceptual model—introduced at the outset of this review and used as its organizing structure throughout—emphasizes that infection-associated psychiatric manifestations should be viewed as dynamic neuroimmune processes rather than isolated disease-specific entities. Although substantial progress has been made in identifying candidate biomarkers, their greatest clinical value will likely derive from multimodal approaches integrating molecular, imaging, and clinical data.

Translating this biomarker-based framework into practice, precise mechanism-based approaches are beginning to emerge in the form of repurposed immunomodulatory agents—such as minocycline, IL-6 receptor blockade with tocilizumab, and COX-2 inhibitors—currently under clinical investigation for post-infectious and inflammation-related psychiatric symptoms. Future studies should focus on validating the proposed integrated model, including its neurotrophic component, in longitudinal cohorts to facilitate precision diagnostics and mechanism-based therapeutic strategies for infection-associated psychiatric disorders.

By integrating diverse infectious diseases, neuroimmune mechanisms, and biomarker domains—including neurotrophic signaling—into a single conceptual framework, this review provides a foundation for a more biologically informed and transdiagnostic understanding of infection-associated psychiatric disorders. Beyond synthesizing the available evidence, the proposed framework highlights common biological pathways that may help bridge the gap between basic neuroimmunology and clinical psychiatry. Such an approach may facilitate the transition from symptom-based classification toward mechanism-driven precision psychiatry, ultimately supporting earlier risk stratification, personalized therapeutic strategies, and improved long-term patient outcomes.

## Figures and Tables

**Table 1:** Representative infectious disease models associated with neuropsychiatric manifestations: pathophysiological mechanisms, neurobiological targets, and clinical phenotypes.

Infectious Agent	Pathophysiological Mechanism	Neurobiological Targets	Neuropsychiatric Manifestations	Ref.
Sepsis (systemic)	Pro-inflammatory cytokine release (IL-1β, IL-6, TNF-α), BBB dysfunction, and neuronal metabolic dysfunction	Diffuse cortical networks, BBB endothelium	Delirium, coma, cognitive dysfunction	[15]
Neisseria meningitidis	Acute meningeal inflammation and BBB dysfunction	Meninges, cerebral endothelium	Delirium, confusion, persistent cognitive sequelae	[12]
Salmonella Typhi	Toxic–metabolic encephalopathy and systemic cytokine response	Cerebral cortex, diffuse cortical networks	Delirium, apathy, confusion	[12]
Treponema pallidum	Cerebral vasculitis and chronic neuroinflammation	Cerebral cortex, cerebral vasculature	Dementia, psychosis, personality changes	[15]
Borrelia burgdorferi	Persistent neuroinflammation	Cortical networks, peripheral nerves	Depression, anxiety, cognitive dysfunction	[15]
Mycobacterium leprae	Peripheral neuroinvasion and chronic inflammation	Peripheral nerves	Secondary affective disorders	[15]
Rabies virus	Retrograde neuronal transport and limbic involvement	Limbic system (amygdala, hippocampus)	Agitation, acute psychosis, delirium	[15]
SARS-CoV-2	Systemic neuroinflammation, endotheliitis, BBB dysfunction, and cerebral microthrombosis	Cerebral endothelium, microglia, prefrontal cortex	Delirium, anxiety, depression, brain fog	[15]
West Nile virus	Neurotropism and neuronal inflammation	Cortex, hippocampus, brainstem	Delirium, memory impairment	[12]
HSV-1	Neuroinvasion, limbic encephalitis, and local neuroinflammation	Hippocampus, medial temporal lobe, microglia	Acute psychosis, delirium, hallucinations, cognitive impairment	[12]
HIV	Chronic microglial activation and viral neurotoxicity	Microglia, white matter, fronto-subcortical networks	HAND, depression, anxiety, cognitive impairment	[15]
Plasmodium falciparum	Cerebral microangiopathy, hypoxia, and microcirculatory dysfunction	Cerebral endothelium, microcirculation	Severe delirium, coma, cognitive impairment	[12]
Trypanosoma brucei	Neuroinvasion and circadian rhythm disruption	Thalamus, thalamo-cortical networks	Sleep disturbances, confusion, psychosis	[15]
Toxoplasma gondii	Cerebral cyst formation, dopaminergic dysregulation, and chronic neuroinflammation	Basal ganglia, dopaminergic circuits	Psychosis, anxiety, behavioral changes	[15]
Taenia solium	Cystic brain lesions, local inflammation, and mass effect	Cerebral cortex, cerebral ventricles	Epilepsy, cognitive impairment, depression	[15]
Cryptococcus neoformans	Chronic meningitis and intracranial hypertension	Meninges, choroid plexus	Confusion, apathy, delirium	[8]

## Data Availability

No new data were created or analyzed in this study. Data sharing is not applicable to this article.

## Abbreviations

AIDS—acquired immunodeficiency syndrome; ART—antiretroviral therapy; ASD—autism spectrum disorder; BAFF—B-cell-activating factor; BBB—blood–brain barrier; BD—bipolar disorder; BDNF—brain-derived neurotrophic factor; C—complement; CNS—central nervous system; COVID—coronavirus infectious disease; CRP—C-reactive protein; CSF—cerebrospinal fluid; DA—dopamine; DAMPs—damage-associated molecular patterns; DTI—diffusion tensor imaging; EBV—Epstein–Barr virus; GFAP—glial fibrillary acidic protein; HAND—HIV-associated neurocognitive disorders; HERVs—human endogenous retroviruses; HPA—hypothalamic–pituitary–adrenal axis; HRV—heart rate variability; HSV—herpes simplex virus; HIV—human immunodeficiency virus; IFN—interferon; IL—interleukin; IDO—indoleamine 2,3-dioxygenase; LPS—lipopolysaccharide; MAPK—mitogen-activated protein kinase; MCP—monocyte chemoattractant protein; MIA—maternal immune activation; MIP—macrophage inflammatory protein-1 beta; MMP—matrix metalloproteinase; MRI—magnetic resonance imaging; NGF—nerve growth factor; NMDA—N-methyl-D-aspartate; NF-κB—nuclear factor kappa-light-chain-enhancer; NfL—neurofilament light chain; PAMPs—pathogen-associated molecular patterns; PET—positron emission tomography; PRRs—pattern recognition receptors; PI3K/Akt—phosphoinositide 3-kinase (PI3K) phosphorylates and activates AKT; PTSD—post-traumatic stress disorder; ROS—reactive oxygen species; rsFC—resting-state functional connectivity; SCZ—schizophrenia; SCFA—short-chain fatty acid; sTREM2—soluble triggering receptor expressed on myeloid cells 2; TNF-α—tumor necrosis factor alpha; TJ—tight junction; TLR—Toll-like receptor; TRP/KYN—tryptophan/kynurenine ratio; TSPO—translocator protein; ZO—zonula occludens; WHO—World Health Organization.
